# The Metal in My Body: Patients’ Perception and Attitude Toward Orthopedic Implants

**DOI:** 10.7759/cureus.56493

**Published:** 2024-03-19

**Authors:** Eleni Tsalkitzi, Dimitrios Kitridis, Elena Heinz, Christina Hionidou, Kornilia Givissi, Panagiotis Givissis

**Affiliations:** 1 Psychiatric Department, 424 Army General Training Hospital, Thessaloniki, GRC; 2 Orthopedic Department, School of Medicine, Aristotle University of Thessaloniki, Thessaloniki, GRC; 3 Orthopedics and Trauma Department, 424 Army General Training Hospital, Thessaloniki, GRC

**Keywords:** orthopedic implants, body image, implant acceptance, injury, fracture

## Abstract

Introduction: Metal implants are broadly used in orthopedics and traumatology to stabilize bone fragments. This study aimed to explore patients’ awareness, body image, and overall experience of living with a metal implant after a fracture.

Methods: A mixed methods convergent design (QUAN+QUAL) was adopted. A self-reported 30-item questionnaire was used to investigate patients’ perception and apprehension of the implantation of orthopedic materials. To enlighten the quantitative findings, semi-structured interviews followed till data saturation. Quantitative and qualitative data were compared during the analysis phase.

Results: Results showed that women’s and elders’ acceptance of the implants was greater than that of men and younger patients even in acute cases. The sense of superiority provided by the implant was mainly reported by the elderly (adjusted odds ratio (OR_adj_) for increasing age: 1.06; 95% CI: 1.02-1.1; p<0.01), and the sense of inferiority was mainly reported by young men (OR_adj_: 6.19; 95% CI: 2.36-16.22; p<0.01). Similarly, women and elderly mostly tended to answer that the injured limb felt stronger after the implant placement, while young men tended to answer a sense of weakness with the implant (OR_adj_ for increasing age: 1.06; 95% CI: 1.03-1.09; OR_adj_ for male gender: 4.67; 95% CI: 1.87-11.7; p<0.01 for both regressions). Most participants (56.6%) and mainly young participants, regardless of gender, expressed the desire to get the metal implants removed (OR_adj_ for increasing age: 0.91; 95% CI: 0.89-0.95; p<0.01). Misinformation and misconception were also found in a high percentage of the questioned patients (48.1%). Thematic analysis of the interviews revealed that none of the participants directly attributed any change in their life, self, or body image to the implants. An altered body image was not reported. The most reported experience was the restriction of movement due to the accident or the subsequent physical weakness.

Conclusion: Despite the acceptance of the implant being great, the level of patient knowledge was fairly low. The present study highlights the importance of providing patients with information throughout their management to avoid misunderstandings. Age and gender did influence patients’ perception of the implants. Personalized assessment is further needed to address body image issues after an implant placement procedure.

## Introduction

Fractures are the most commonly occurring forms of trauma with a global incidence of 9.0-22.8/1000/year, affecting mostly young men and older women [1,2]. Unstable fractures that can not be treated with a cast or splint require stabilization with an external fixator or open reduction and internal fixation (ORIF). Internal fixation is a surgical procedure used to internally set and stabilize fractured bones. During the procedure, the bone fragments are first repositioned into their normal alignment and then held together with special implants, such as plates, screws, nails, and wires. Stainless steel, cobalt-chrome (CoCr) alloys, and titanium (Ti) alloys are commonly selected for the manufacturing of these implants [3].

Surgical removal of the hardware after the bone union is a frequently performed orthopedic surgery. In a retrospective study conducted by Reith et al. (2015), 30% of the participants (N=332) wanted the metal implant to be removed for personal reasons and 24% reported foreign body sensation as the reason for the removal decision. These findings indicate that, apart from the biological response to the metal implant (allergy, infection, nerve damage, etc.), the psychological response matters. Issues like foreign body sensation and subjective pain may emerge, affecting the patient’s experience and attitude toward the metal implant.

To facilitate the rehabilitation process, healthcare professionals must understand patients’ responses to this body change. Similarly, patients’ understanding of their medical problems is integral to their ability to make competent decisions, comply with treatment, and enable recovery. As the literature indicates, the level of patient knowledge and attitude toward orthopedic metal implants is poorly known [3-5].

To our knowledge, no study has been carried out, to date, examining the perception and experience of patients with a metal implant in their body after a fracture. Two research questions were posed: 1) what do patients know about their implants? and 2) How do they experience living with a metal implant inside their body?

## Materials and methods

Study design and participants 

A prospective single-center study was conducted after Institutional Review Board (IRB) approval was obtained (162/23.11.2015). A mixed-method approach was chosen. The study included two rounds of data collection. Round 1 consisted of a questionnaire-based survey, and round 2 included individual interviews. A purposive sampling technique was applied to recruit participants. Adult consecutive patients who had been subjected to an implant placement procedure after a fracture in the Orthopedic Department of a University hospital from 2016 to 2019 were included in the study. Exclusion criteria were as follows: age younger than 18 years, guardianship, impaired cognition or memory, presence of implant-related complication, removal of the external or the internal fixator, lack of fluency in written and/or spoken Greek, subjects with an incomplete survey, and patient refusal to participate in the study. All participants viewed a post-operative radiograph to facilitate visualization of their internal body with the object, after the implant surgery.

Data collection 

Round 1

Round 1 consisted of a self-reported questionnaire sent by mail to a random sample of eligible patients. The questionnaire included 30 items covering five domains (socio-demographic information, knowledge about the implant, the post-placement experience, compliance with the treatment, and general satisfaction), and was developed by the authors for this study. The items had single- or multiple-choice questions. A box was provided to specify affirmative replies. The questionnaire was set in a generally understandable format, in Greek, avoiding the use of technical terms. A letter was also attached that described the study’s purposes and the confidentiality of the responses. Patients were asked to answer the questionnaire alone, without the aid of an interpreter. 252 questionnaires were sent in total. 

**Figure 1:**
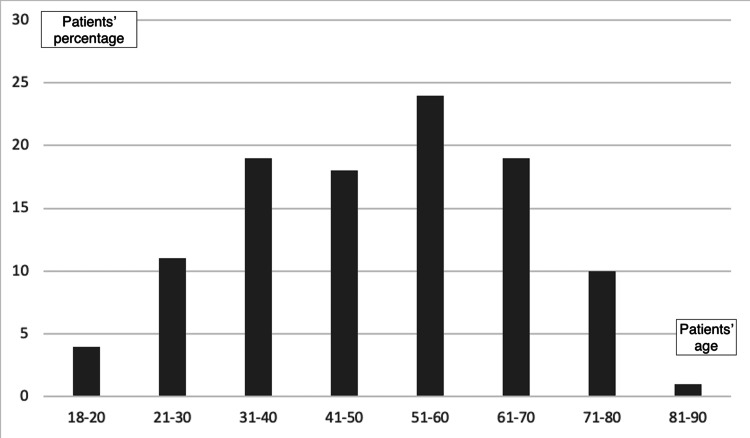
Participants' distribution by age

A total of 106 responses were received, corresponding to a 42.1% response rate. The mean age of the participants was 49.2±16.1 years (47.2% males, 52.8% females). Most of them were married (66%) and had primary education (51%). Few participants held an external fixator (17%). The time elapsed since the implant placement procedure ranged from six months to three years. Table 1 and Figure 1 show their detailed characteristics.

**Table 1:** Participants' characteristics

Variables	Round 1 (N=106)	Round 2 (N=18)
Age (years)		
18-20	4	1
21-30	11	4
31-40	19	5
41-50	18	2
51-60	24	2
61-70	19	3
71-80	10	1
81-90	1	0
Gender		
Male	50	11
Female	56	7
Localization of the implant		
Upper limb		
Hand	2	1
Forearm	4	1
Arm	1	2
Elbow	2	1
Lower limb		
Hip	48	6
Femur	14	2
Leg	17	2
Ankle	13	2
Knee	5	1
Time since the surgery		
6-12 months	31	5
13-18 months	45	7
19-24 months	22	3
>24 months	8	3

SPSS software package was used for analysis. The replies were presented as percentages and illustrated with simple graphs. Logistic regression was used to model the association between selected replies and demographic characteristics. Adjusted multivariable logistic regression to utilize for eliminating confounding factors. 

Round 2

Round 2 was added to enlighten the findings of the quantitative survey that took place in round 1. Round 2 consisted of 18 semi-structured interviews. The sample of the qualitative strand was different from the sample of the quantitative strand, but the inclusion criteria were the same (Table 1). The sample size was determined based on the data saturation criterion applied in the data collection of qualitative studies. All interviews were conducted face-to-face and were based on a 13-item pilot-tested interview guide (Table 2). The questions were open-ended and designed to explore knowledge and experience after the implant placement operation. Interviews took place in a private doctor’s office in the Orthopedic Department of the hospital and lasted about 40 minutes. Before the interview, participants were asked to fill in a demographic sheet and sign the informed consent. Permission to audiotape the interviews was also obtained. 

**Table 2:** Semi-structured interview questions

Interview guide
What do you know about the implant? (material, function, the way it adheres to the bone, external appearance)
How would you describe the implant to someone that has no idea about implants?
Do you know if the implant/material can cause problems?
What were your first concerns about the implant?
What concerns do you now have? about the implant?
Have you ever experienced any problems with the implant?
How did you gain information about the implant/material?
How do you feel about having the implant?
Do you know if the implant/material has any benefits?
Do you feel your body differently compared to before? How do you feel your body now?
How do you feel in relation to others?
Has your behavior changed compared to before? How?
What do you expect as soon as you have the implant removed?

Thematic analysis was applied to analyze the transcribed interview data. After familiarizing with the data, a search for common themes took place. The themes and patterns that emerged during the analysis were identified. Modifications were made when a new category did not fit the pre-established ones. The two authors (first and second) coded the narratives independently and separately. The third author interfered in cases of disputes. Numbers were used instead of patients’ names to ensure anonymity. 

To ensure the integrity of the qualitative data, we adopted Lincoln and Guba’s evaluative criteria [6]. Peer debriefing and member checking were used, and patients’ authentic quotes were included in the writing of the final report. 

## Results

Results round 1

**Figure 2:**
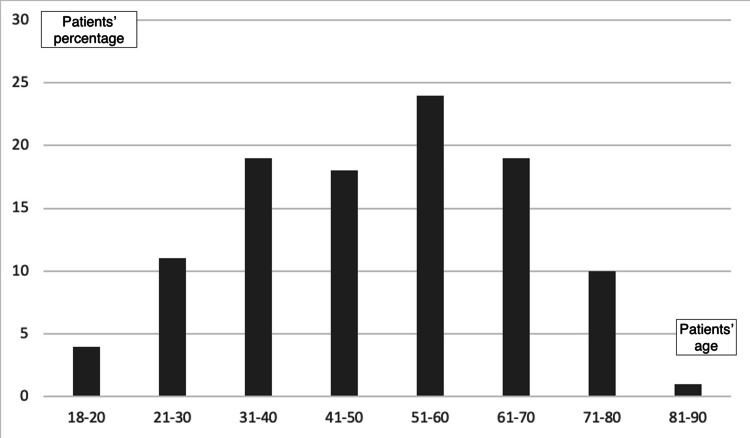
Participants' distribution by age

Analysis of the 106 completed questionnaires revealed ignorance and indifference about the metal implant in their body. Nearly half of the participants (48.1%) did not know the kind of material that the implant was made of, and 21.7% of the participants answered falsely in this question (Figure 2). Among their answers were the following: gold, platinum, airplane materials, silver, plastic, porcelain, and bronze. Participants between the ages of 30 and 40 years demonstrated better knowledge about the implant than participants in other age groups.

When asked about information provision, 54.7% of the patients said that they preferred to look for information about their implant on co-sufferers in their familiar environment and on websites rather than on the television, in magazines, or in newspapers. None of the participants had researched press reports about their implants. Yet, the information they had acquired was referring to possible complications and not technical properties. 

**Figure 3:**
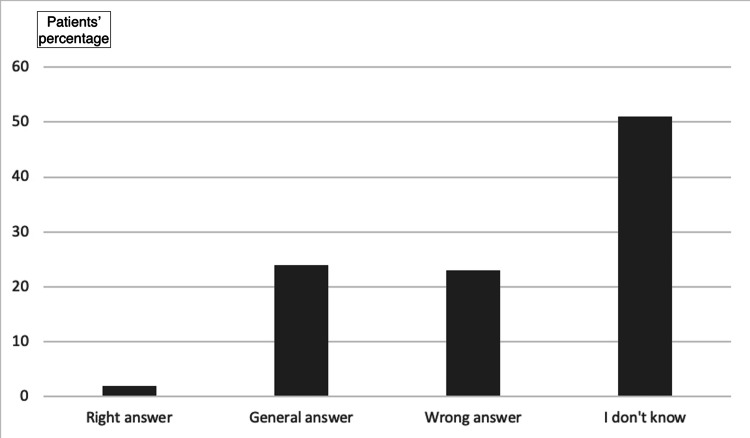
Knowledge about the material of the implant

**Figure 4:**
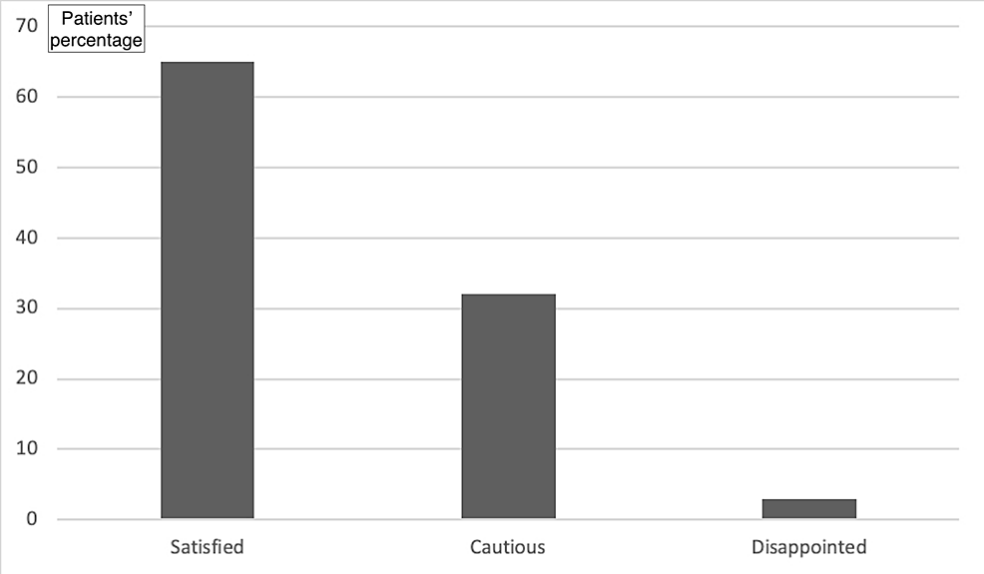
Overall satisfaction with the implant placement

Despite their ignorance about their implants, most of the participants recognize the implants’ usefulness in orthopedics and trauma. In the question “Would you prefer if implants hadn’t been used?” 34% of the participants answered “no,” while 13.2% answered “yes.” The remaining 52.8% answered “I don’t know.” Additionally, most participants (60.4%) reported a personal belief that the implant does not cause any problem, compared to the remaining 39.6% reporting the opposite belief. Generally, most of the participants (61.3%) were satisfied with the treatment with the implant employment (Figure 3). Nevertheless, most participants (56.6%) and mainly young participants regardless of gender expressed the desire to get the metal implants removed (adjusted odds ratio (OR_adj_) for increasing age: 0.91; 95% CI: 0.89-0.95; p<0.01; OR_adj_ for gender: 0.98; 95% CI: 0.38-2.53; p=0.97; Figure 4).

**Figure 5:**
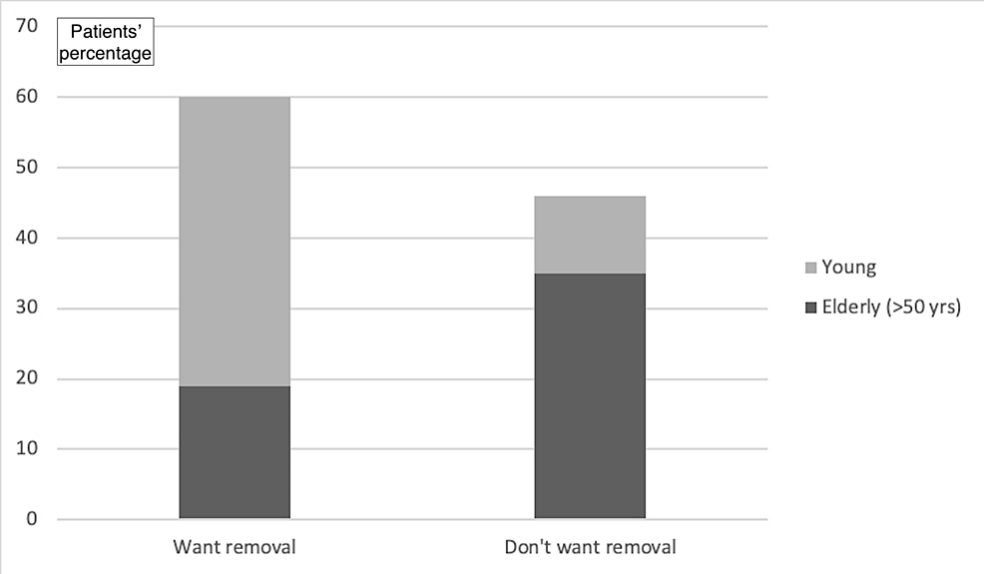
Thoughts about implant removal

**Figure 6:**
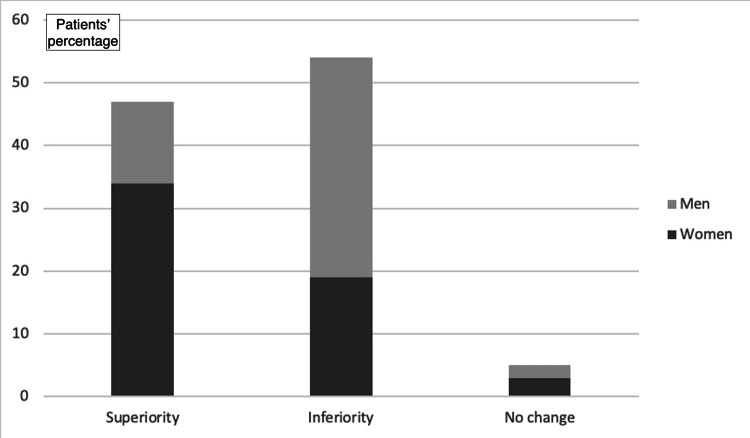
Impact on the sense of superiority after implant placement

As far as the sense of superiority or inferiority that the implant gives, 50.9% reported a sense of inferiority and 44.3% reported a sense of superiority, while 4.7% reported no change (Figure 4). The sense of superiority was mainly reported by the elderly (OR_adj_ for increasing age: 1.06; 95% CI: 1.02-1.1; p<0.01), and the sense of inferiority was mainly reported by young men (OR_adj_: 6.19; 95% CI: 2.36-16.22; p<0.01; Figure 5 and Figure 6). 

**Figure 7:**
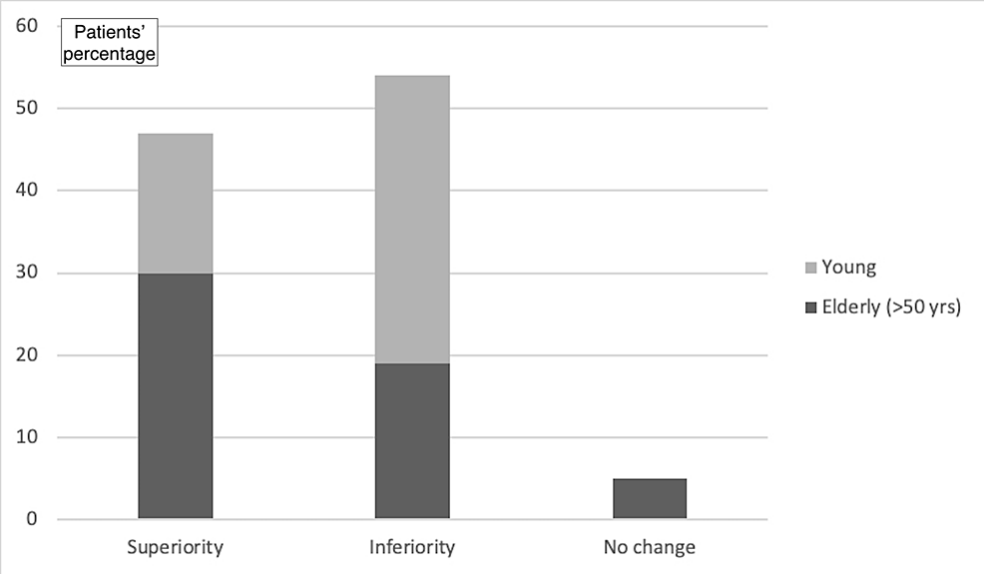
Impact on the sense of superiority after implant placement (based on participants' age)

**Figure 8:**
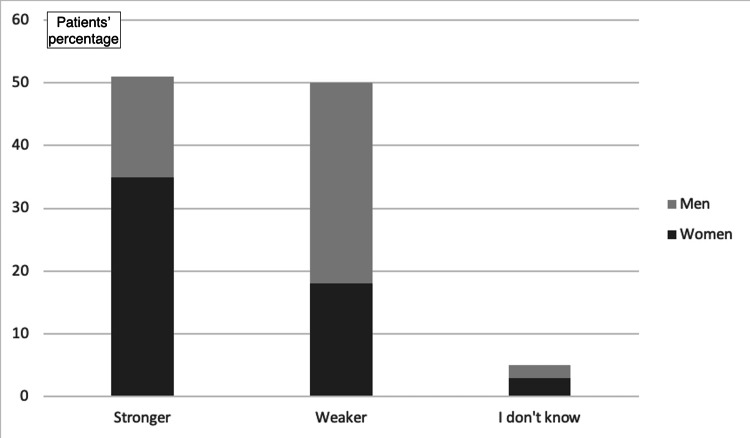
Impact on the sense of power after implant placement

**Figure 9:**
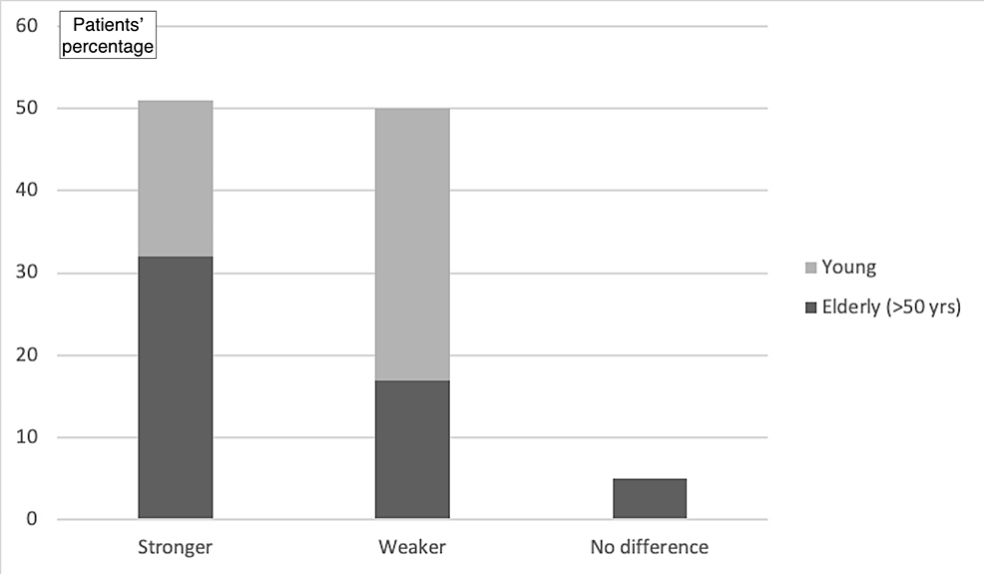
Impact on the sense of power after implant placement (based on participants' age)

47.2% of the participants revealed that they felt the injured limb was stronger with the implant, whereas 48.1% of the participants stated that they felt the limb was weaker. The remaining 4.7% reported no difference. Following adjustment for demographic data, it was found that women and the elderly mostly tended to answer that the injured limb felt stronger after the implant placement, while young men tended to answer a sense of weakness with the implant (OR_adj_ for increasing age: 1.06; 95% CI: 1.03-1.09; OR_adj_ for male gender: 4.67; 95% CI: 1.87-11.7; p<0.01 for both regressions; Figure 7, Figure 8, and Figure 9). 

When asked about the influence of implant visibility, 34.9% of the participants answered that, if the implants were visible, it would affect their behavior more negatively. 

No major changes in the work environment and interpersonal relationships were reported by the participants in this data collection round. In detail, 69.8% of the participants reported that their family’s and friends’ behavior toward them was normal, and only 30.2% faced a change in their work referring to temporary absence due to their medical condition. To enlighten and expand these findings, semi-structured interviews followed in the next round. 

Results round 2

Four key themes emerged from the 18 transcribed interviews: “No questions about the implant please. I don’t know and I actually don’t even care,” “The accident changed me and my life,” “Adaptation and acceptance,” and “A trustworthy doctor-patient relationship is vital.”

Theme 1: “No Questions About the Implant, Please. I Don’t Know and I Actually Don’t Even Care”

Only three out of 18 knew the implant’s name and seven out of 18 knew some of the implant’s characteristics/properties. Also, only four participants talked about advantages (quick movement restoration and protection against infections) and only five participants reported disadvantages (three reported infection risk and two reported pain). Although the participants revealed that they had sought advice about the implants, the given advice focused mainly on the possible risks rather than on descriptive facts about the implants. Interestingly, most participants (10/18) sought advice from co-sufferers, reporting that the co-sufferers’ advice was mostly deterring. Yet, this deterrent information did not negatively affect their positive attitude toward the implant. As one participant characteristically said “I faced them with caution. Each one is a unique situation, and I am not a doctor to know.” In a similar thread, only four participants had concerns about the implant before the surgery. These concerns regarded the fear of an infection or the fear of repetitive surgery. 

Participant 2: “I was worried about an infection. I have heard that there is such a possibility.” 

Participant 11: “I worried whether the implant would be internal or external. I have this personal belief that the external implant is more effective.”

Participant 14: “I wanted the implant to be external. I did not want to get into the surgery process again by removing an internal implant.”

Participant 15: “A friend of mine suffered an infection due to the implant. I would not want this to happen to me.”

At the end of the interview, 15 out of 18 participants noted their ignorance about the implant that was revealed during the interview. A participant mentioned “Look what has happened here. Eventually there were so many things about the implant that I had not asked,” and another one said “I am surprised to find out that I did not actually care to know about the implant.” 

Theme 2: “The Accident Changed Me and My Life”

Most of the participants reported a change in their life and sense of self after the accident and the implant surgery. Within this theme, three subthemes were apparent from the interviews: “Lifestyle modifications,” “My body feels different somehow,” and “Behavioral changes.” 

Subtheme 1: Lifestyle Modifications 

Transition to a nonactive lifestyle was reported by most of the participants. Fear of movement was evident in their narratives. Most participants explained that after the accident their everyday activities got restricted. They preferred to stay at home and use the elevator.

Participant 1: “I do not climb stairs because I am afraid that I will fall, and the screws will come out.” 

Participant 4: “I prefer online shopping… The pavements aren’t so safe and with all the chuckholes I don’t want to fall and the implant break.” 

Participant 7: “I don’t run for long distances like I used to. I try to be in shape without pushing myself too hard.”

Participant 11: “I mostly stay at home. I sometimes walk carefully around the neighborhood. I avoid using my car.”

Interestingly, none of the participants holding an internal implant attributed the life change to the implant directly. The change was rather due to the accident and the fear that by being clumsy the implant would break or get dislocated. As a participant stated clearly “My life changed after the accident. I don’t blame the implant for the change. The implant was a necessity.”

On the other hand, five participants holding an external fixator tended to directly link some life changes to the implant. As they stated, the visibility of the implant was the main reason to avoid socializing and feeling “sick” and dependent. 

Participant 9: “I don’t want to socialize. I don’t want other people staring at me.”

Participant 12: “I depend on others. I can’t do much with this on my leg. I am feeling disabled.” 

Participant 15: “As long as I have this, I can’t help but feeling sick.” 

A participant revealed applying a “camouflage technique” even when being on her own. 

Participant 5: “I don’t want to look at the external fixator. I wear long skirts to hide it even when I am being alone in the house.” 

*Subtheme 2: My Body Feels Different Somehow* 

Almost all the participants (15/18) reported a sense of a “different” body after the surgery. Most participants talked about a change in body strength and a feeling of pain/heaviness in their daily body movements. The body changes were not associated with the implant itself but with the general physical condition.

Participant 6: “I used to be strong, but now I am not. I may be afraid to put strength."

Participant 8: “I am afraid to cross the streets because I have difficulty running.”

Participant 13: “I feel my body heavier. Maybe I am physically weaker.” 

Participant 16: “I feel the same from my waist and above, but from my legs and below I feel my body different because it is different."

Participant 9: he was feeling weaker only at the beginning while he had the crunches: "I felt disadvantaged at first, that I lost something of my strength, less capable."

A positive body change was reported by one participant, who was the only one declaring that he felt more secure with the implants in his body: "Because I do not know if I can trust the bone as a bone, I feel safer that the implants exist" (Participant 15).

Subtheme 3: Behavioral Changes

Half of the participants revealed a change in their behavior. Tantrums, autonomy, strength loss, and a change in the way of thinking about life were among the reported changes. 

Participant 5: “I became more introverted because of the accident. No change from the implant."

Participant 7: “I am closer to my family now. After the accident, I decided to put my energy into the important people in my life.”

Participant 10: “I feel more agitated. Not because of the implant but because I have to change my life and restrict my activities.” 

A participant made a clear distinction: “I don’t feel my body different; my soul feels different. It’s like a new, more fragile “me”.

*Theme 3: Acceptance and Adaptation* 

Most participants (13/18) had a positive attitude toward the implant. They were neither talking negatively about it, nor feeling it was a foreign body inside them, and did not want to get it removed. 

Participant 2: “I don’t think about it. So many people have their screws!”

Participant 12: “I went to the airport and was disappointed that the alarm wasn’t activated hahaha. I live with them; They have become part of my life.”

Participant 17: “I don’t mind taking them off. It makes no difference to me.”

Four of these participants described a gradual process to the final stage of accepting the implant. In the beginning, the crutches, the pain after the surgery, and the adaptations needed in the daily routine made it difficult for them to smoothly accept the implant with no cost. Agitation, grief, hesitation, and fears were experienced in these first stages, after the implant placement. 

Participant 3: “As long as I had the crutches, I was more irritable. Then I settled down.”

Participant 10: “The first days were rough. I was afraid to move my leg. I was feeling it stiff. I needed so time to adapt.”

Participant 16: “The adjustment wasn’t easy. I wanted to be just like before the accident. I gradually acknowledged that there is no point to dwell in the past.”

Only five young male participants stated the need to get the implants removed. 

Participant 1: “I feel like I have something foreign in my leg, psychologically I mean. I want to be just like before.”

Participant 18: “I always worry when I will take it off.”

Theme 4: A Trustworthy Doctor-Patient Relationship Is Vital

A trustworthy doctor-patient relationship was considered vital by all participants. 

Most participants (10/18) justified their ignorance about the implant by their trust in their doctor and the medical community. One of them clearly stated, “I trust my doctor and I trust that this treatment was the best for me.”

It is interesting to note that, even though the majority reported that they had complied with the doctor’s instructions, seven out of 18 participants (22%) said that they were not following the instructions provided completely. 

Participant 9: “In the first month post-surgery I was very careful and followed the doctors’ instructions faithfully. Then I kind of loosened up.”

Participant 14: “As the pain subsided, I felt more confident. Gradually I stopped considering my doctor’s instructions.” 

Participant 18: “There is this rehabilitation protocol. I knew what I was supposed to do, but I thought it was too strict and long-term to follow it line by line.”

## Discussion

The current study employed a mixed-method design to explore the patients’ attitudes and knowledge toward their orthopedic implant after the placement procedure. Results revealed that patients with orthopedic implants tend to accept the metal element in their body and not acknowledge it as a factor directly influencing their identity, body image, and life. This does not mean that changes did not occur. The fracture affects the patient’s life on many levels. Participants talked about incomplete functional restoration, movement restrictions, fear of re-injury, dependence, and pain even after bone stabilization. The adaptation process involved lifestyle modifications. Several participants described transitioning from a fear of movement to a more confident way of moving over time. In both rounds, elderly and female participants tended to accept the implant better and make more positive statements regarding life afterward. Moreover, the sense of superiority was mainly reported by the elderly (OR_adj_ for increasing age: 1.06; 95% CI: 1.02-1.1; p<0.01), and the sense of inferiority was mainly reported by young men (OR_adj_: 6.19; 95% CI: 2.36-16.22; p<0.01). Women and elderly mostly tended to answer that the injured limb felt stronger after the implant placement, while young men tended to answer a sense of weakness with the implant (OR_adj_ for increasing age: 1.06; 95% CI: 1.03-1.09; OR_adj_ for male gender: 4.67; 95% CI: 1.87-11.7; p<0.01 for both regressions). 

Although the acceptance of the orthopedic implant is great, the relevant knowledge is low. Misconceptions and misunderstandings about the implant were common among the participants. Their many “I don’t know” answers indicate a degree of indifference about the implant, a fact that reveals that the implant did not seem to preoccupy them a lot. This trust in the implants derives from the trust in the doctor and their approval by the medical community, as most participants explained. Comparing the results of the two rounds, ignorance about its properties (48.1%) is evident (material, advantages and disadvantages, risks, alternative implant options, name). Participants aged between 30 and 40 years demonstrated better knowledge about the implant than participants in other age groups. In both rounds, the main source of information about the implant was the co-sufferers. These findings are consistent with those of Abu Al-Rub, Hussaini, & Gerrand (2014). The above authors examined the level of understanding among patients who had undergone joint replacement and found that the majority of them did not know what type of joint replacement they had and what materials the implant was made of [4]. Previous studies have demonstrated similar shortcomings in patient comprehension in both emergency department and orthopedic settings [5,7-10]. Orthopedic patients frequently misunderstand common orthopedic terminology and understand little about their injury, acute management, and ongoing treatment [7,10]. In their study, Schiller et al. (2015) concluded that patients had little knowledge about their hip fracture (i.e., available resources, expected recovery process) and similarly, in a study conducted by Kampa, Pang, & Gleeson (2006), most participants wrongly believed that there was a difference between a "fracture" and a "break." 

The majority of the study participants (61.3%) reported a desire to get the implant removed. Young patients seem to mostly express the need for removal (OR_adj_ for increasing age: 0.91; 95% CI: 0.89-0.95). The same desire was found in the study by Reith et al in 2015, where 30% of the study participants expressed a personal preference to have the metal implant removed [11]. Interestingly, in the qualitative study conducted by Watson, Martin, & Keating (2018), most patients submitted to surgery after a wrist fracture reported a sense of relief when the cast was removed [12]. 

Regarding implant visibility, 34.9% of the participants answered that if the implants were visible, it would affect their behavior more negatively. A small percentage of the participants held an external fixator (17%). These participants reported a feeling of minority compared to other people, a dependence on others in their daily movements, a “camouflage” technique to hide the visible implant, and a drop in their self-esteem. Their answers are in line with findings reported by other studies [13-16]. Altered body image was not found in these studies either. Concerning the work environment and interpersonal relationships, only 30.2% of the participants faced a change in their work referring to temporary absence due to their medical condition, and 69.8% of the participants reported that their family’s and friends’ behavior toward them was normal.

There are several qualitative studies evaluating the impact of implant placement in orthopedic patients. Yet, no study emphasized the implant itself. Most studies examined the pre-surgery or post-surgery quality of life rather than the position that the implant holds in the patient’s mental world. These studies revealed the same changes that the present study’s participants reported; lack of independence, isolation, incomplete recovery, and pain after acute fracture stabilization and the bone union had occurred [17-20]. Similarly, a large retrospective study examining long-term outcomes (>2 years) after operative repair of pilon tibial fractures found impaired functionality and lower health-related quality of life (HRQoL score) compared to an uninjured reference population [21]. 

Inconvenience in airport flights was not reported by the participants. The fact that orthopedic implants are detected by metal detectors in airports is well-recognized in the literature [22-25]. When asked, patients with a metal implant stated that an implant identification card outlining what kind of implant they possessed would be very useful at airport checks [26]. 

Limitations of this study include a lack of diversity in the final sample, the use of a non-validated questionnaire, and the retrospective nature of the study design. The participant’s perception of their experience may be different if the data collection had occurred chronically closer to the implant placement procedure. Also, the sample size in round 2 of the study may seem relatively small but is based on the data saturation criterion that is applied in the sampling of qualitative studies. Functional outcomes also were not recorded as the aim of the study was to evaluate the subjective psychological experience. 

Despite these drawbacks, the study is the first of its kind, providing data about orthopedic implants from the patient perspective. Similar studies using quantitative or qualitative measures to expand the findings of the present study are warranted. Further studies that include a larger group from various socioeconomic/racial backgrounds would help to confirm these initial findings. An additional recording of functional or HRQoL outcomes would also add to the understanding. 

## Conclusions

The implant does not seem to preoccupy the participants physically or mentally. The majority of the studied patients accepted the implant and recognized it as a facilitator in the journey toward functional restoration. Although the acceptance of the implant was great, the level of patient knowledge was fairly low. Personalized assessment is further needed to address potential body image issues after an implant placement procedure. Women and the elderly mostly felt that the injured limb felt stronger after the implant placement, while young men tended to report a sense of weakness with the implant. Our study aimed to offer an initial insight into the patient’s perspective on orthopedic implants used in internal fixation surgeries, providing a basis for further analysis, repetition, and confirmation, or not, of the study findings.
